# Time course of airway remodelling after an acute chlorine gas exposure in mice

**DOI:** 10.1186/1465-9921-9-61

**Published:** 2008-08-14

**Authors:** Stephanie A Tuck, David Ramos-Barbón, Holly Campbell, Toby McGovern, Harry Karmouty-Quintana, James G Martin

**Affiliations:** 1 Meakins-Christie Laboratories, McGill University, Montreal, Canada; 2 Complejo Hospitalario Universitario Juan Canalejo, A Coruña, Spain

## Abstract

Accidental chlorine (Cl_2_) gas inhalation is a common cause of acute airway injury. However, little is known about the kinetics of airway injury and repair after Cl_2 _exposure. We investigated the time course of airway epithelial damage and repair in mice after a single exposure to a high concentration of Cl_2 _gas. Mice were exposed to 800 ppm Cl_2 _gas for 5 minutes and studied from 12 hrs to 10 days post-exposure. The acute injury phase after Cl_2 _exposure (≤ 24 hrs post-exposure) was characterized by airway epithelial cell apoptosis (increased TUNEL staining) and sloughing, elevated protein in bronchoalveolar lavage fluid, and a modest increase in airway responses to methacholine. The repair phase after Cl_2 _exposure was characterized by increased airway epithelial cell proliferation, measured by immunoreactive proliferating cell nuclear antigen (PCNA), with maximal proliferation occurring 5 days after Cl_2 _exposure. At 10 days after Cl_2 _exposure the airway smooth muscle mass was increased relative to controls, suggestive of airway smooth muscle hyperplasia and there was evidence of airway fibrosis. No increase in goblet cells occurred at any time point. We conclude that a single exposure of mice to Cl_2 _gas causes acute changes in lung function, including pulmonary responsiveness to methacholine challenge, associated with airway damage, followed by subsequent repair and airway remodelling.

## Introduction

Chlorine (Cl_2_) gas is a common inhalational irritant, encountered both occupationally and environmentally[1,2]. The acute effects of Cl_2 _gas inhalation can range from mild respiratory mucus membrane irritation to marked denudation of the mucosa, pulmonary oedema, and even death. Recovery from Cl_2_-induced lung injury requires repair and/or regeneration of the epithelial layer. The repair process after Cl_2 _exposure may not restore normal structure and function as cases of subepithelial fibrosis, mucous hyperplasia, and non-specific airway hyperresponsiveness have been reported in persons after recovery from Cl_2 _injury[3,4]. Repeated exposure to chlorine through swimming appears to be a significant risk factor for airway disease manifesting as asthma[5].

The airway epithelium is the first target of inhaled Cl_2 _gas. Although the exact mechanism of epithelial damage is unknown, oxidative injury is likely involved as Cl_2 _gas can combine with reactive oxygen species to form a variety of highly reactive oxidants [6]. Direct oxidative injury to the epithelium may occur immediately with exposure to Cl_2_, but further damage to the epithelium may occur with migration of inflammatory cells such as neutrophils into the airway epithelium and the subsequent release of oxidants and proteolytic enzymes.

Limited information is available regarding the time course of injury and repair of the epithelium after acute Cl_2 _gas exposure. Bronchial biopsies from humans have shown epithelial desquamation from 3 to 15 days after accidental Cl_2 _exposure followed by epithelial regeneration, characterized by proliferation of basal cells at two months post-exposure[7]. Animal studies of Cl_2 _exposure have furthered our understanding of the time course of injury and repair. However, these studies have been primarily descriptive in nature. Rats acutely exposed to high concentrations of Cl_2 _gas demonstrated bronchial epithelial sloughing 1 hour after exposure with epithelial regeneration occurring by 72 hrs after exposure[8]. Recently, we have described the response of A/J mice to a single exposure to varying concentrations of Cl_2 _exposure[9]. Exposure to the highest concentration of Cl_2 _gas (800 ppm for 5 minutes) resulted in marked epithelial loss and airway hyperresponsiveness to methacholine 24 hrs after exposure.

Airway remodelling is a feature of asthma that has the potential to explain the induction and chronicity of the disease. Generally animal models have focussed on allergen-driven changes in airway structure which are of uncertain relevance to irritant-induced asthma. For this reason we wished to explore the injury and repair processes involved in irritant-induced asthma. To do this we characterized the time course of airway injury and repair after a single exposure to Cl_2 _gas in mice using quantitative measures of epithelial damage and repair. Markers of epithelial damage were apoptosis, assessed by terminal dUTP nick end labelling (TUNEL) staining, and the presence of protein and epithelial cells in the bronchoalveolar lavage fluid. Epithelial repair was assessed by quantifying cell proliferation using the proliferation marker proliferating cell nuclear antigen (PCNA). PCNA is a DNA polymerase-δ cofactor located in the nuclear compartment of proliferating cells [10,11]. Airway remodelling was assessed by quantification of airway smooth muscle mass using standard morphometric techniques on smooth muscle specific α-actin immunostained tissue sections and by scoring of airway fibrosis on Picrosirius red stained tissue sections. Goblet cell numbers were assessed by light microscopy and standard morphometric techniques. Airway histology was also used to qualitatively assess the time course of damage and repair to the airways. We wished to relate these markers of damage and repair to functional consequences of Cl_2_-induced injury in terms of airway mechanics and airway responsiveness to methacholine.

## Methods

### Animals and chlorine exposure

Male A/J mice (23–27 g) were purchased from Harlan (Indianapolis, Indiana) and housed in a conventional animal facility at McGill University. Animals were treated according to guidelines of the Canadian Council for Animal Care and protocols were approved by the Animal Care Committee of McGill University.

Forty-eight mice were exposed to either room air (control) or 800 ppm Cl_2 _gas diluted in room air for 5 minutes using a nose-only exposure chamber. This concentration of Cl_2 _gas was chosen as it was previously shown to result in severe airway damage but with minimal animal mortality[9]. Mice exposed to Cl_2 _were studied at 12 hrs, 24 hrs, 48 hrs, 5 days (d), or 10 d after Cl_2 _exposure (n = 8 at each time point). The control mice were studied 24 hrs after exposure to room air (n = 8).

### Bronchoalveolar lavage, lung histology and morphometry

The chest was opened, the left main bronchus clamped, and 0.3 ml of sterile saline followed by four separate 0.5 ml instillations were washed into the right lung. Fluid recovered from the first wash was centrifuged at 1500 rpm for 5 minutes at 4°C and the supernatant used for protein quantification. The cell pellet was pooled with the remaining lavage samples and total live and dead cells were counted using trypan blue exclusion. Cytospin slides were prepared using a cytocentrifuge (Shandon, Pittsburgh, PA) and stained with Dip Quick (Jorgensen Labs Inc., Loveland, CO). Differential cell counts, including epithelial cells, were determined on 300 cells/slide. Total protein in the BAL supernatant was quantified using a dye-binding colorimetric assay (Bio-Rad, Hercules, CA), and determined by spectrophotometry at 620 nm and quantified using a bovine serum albumin standard curve.

### Tissue preparation

Following BAL, the lungs were removed and the left lung was fixed with an intratracheal perfusion of 10% buffered formalin at a constant pressure of 25 cmH_2_O for a period of 24 hrs. Histology and immunohistochemistry were performed on 5 μm thick paraffin-embedded sections taken from the parahilar region. Adjacent sections were either stained with hematoxylin-eosin (H&E), periodic acid Schiff (PAS), or processed for immunohistochemistry.

### Immunohistochemistry

Cells undergoing proliferation were detected in tissue sections by immunostaining for proliferating cell associated nuclear antigen (PCNA. Following deparaffination in xylene and rehydration through graded ethanol solutions, the tissue sections underwent a high temperature epitope unmasking treatment by a modified version of the microwave boiling method. An acidic antigen retrieval buffer (Vector Laboratories, Burlingame, CA) was microwave pre-heated to 95°C, and the slides were incubated in it for 30 minutes using a pre-warmed coplin jar protected with styrofoam. After cooling for 20 minutes, a membrane permeabilization treatment was applied by immersing the slides for 20 minutes in a 0.2% dilution of Triton X-100 (Sigma Chemical Co., St. Louis, MO) in pH 7.6 Trizma base (Sigma) buffered saline. The tissues were then blocked for 1 hour using a blocking reagent designed for immunohistochemistry using mouse primary antibodies on mouse tissues (Vector Laboratories). Primary murine anti-PCNA antibody was applied at a concentration of 2.5 μg/ml and the sections were incubated for 30 min. at room temperature. A biotinylated anti-mouse antibody (1:250 dilution; Vector Laboratories) was applied for 10 min. followed by a 45-min. incubation with an avidin-biotin complex-alkaline phosphatase reagent (ABC-AP). Rat intestine was used as a positive control and mouse lung sections incubated with isotype control mouse IgG were used as a negative control. PCNA-positive cells were visualized with Vector Red chromogen (Vector Laboratories) and the tissue was counterstained using methyl green (Sigma). Finally, the sections were dehydrated and mounted under glass coverslips with VectaMount (Vector Laboratories).

To determine the amount of airway smooth muscle by morphometry, airway smooth muscle was detected by immunostaining for smooth muscle α-actin. The lung sections were prepared as described above with the exception of high temperature antigen unmasking, and incubated with monoclonal antibody to smooth muscle α-actin (1A4, 1:1000 dilution; Sigma) for 30 minutes followed by biotinylated anti-mouse IgG antibody and ABC-AP steps as above.

PCNA was colocalized with smooth muscle α-actin in order to detect cell proliferation in the airway smooth muscle. Immunohistochemistry for PCNA was done first as described above, and the signal developed with BCIP/NTB chromogen (Vector Laboratories) instead. The sections were then incubated with anti-smooth muscle α-actin antibody (1A4, 1:1000 dilution, Sigma) for 30 min. at 37°C, followed by the biotinylated anti-mouse antibody and ABC-AP steps as above. The smooth muscle α-actin signal was developed with Vector Red, and the tissues counterstained with methyl green.

### Detection of apoptotic cells *in situ*

To detect apoptotic cells in lung tissue sections we used a TUNEL technique (ApopTag peroxidase detection kit; Intergen, Purchase, NY). The sections were deparaffinized, pretreated with 20 μg/ml proteinase K (Intergen) for 15 min at 37°C, and endogenous peroxidase activity was quenched with 3% hydrogen peroxide for 5 min This was followed by polymerization of digoxigenin-labeled UTP on nicked DNA ends and application of anti-digoxigenin peroxidase conjugate, using ApopTag kit components as per manufacturer's instructions. The signal was developed with DAB chromogen, and the tissues counterstained with methyl green.

### Quantitative morphology on airway sections

Quantification of PCNA-positive cells was performed on parahilar lung sections. Cross-sectioned airways, with a major/minor diameter ratio < 2.5, were selected for analysis. The number of PCNA^+ ^cells in the epithelium and sub-epithelial layers were quantified under a light microscope using a 40× objective. The airway basement membrane length was measured by superimposing the image of the airway onto a calibrated digitizing tablet (Jandel Scientific, Chicago, IL), with a microscope equipped with a *camera lucida *projection system (Leica Microsystems, Richmond Hill, ON, Canada). The numbers of proliferating cells corrected for airway size were expressed as PCNA^+ ^cells/mm of basement membrane perimeter (P_BM_).

### Quantification of ASM mass and proliferation

ASM mass was measured on control, 5 d, and 10 d post-exposure groups by tracing the ASM bundles, as defined by positive staining for smooth muscle α-actin, using a camera lucida and digitizing system. The sum of the ASM bundle areas was calculated for each airway and referenced to P_BM_^2 ^for airway size correction. To determine if airway smooth muscle cells expressed PCNA, co-localization of PCNA with smooth muscle α-actin was done in a subset of animals. The number of PCNA+ cells in the epithelial and sub-epithelial layers of each airway with a major/minor diameter ratio < 2.5 was quantified and expressed per mm of P_BM _for epithelium or P_BM_^2 ^for subepithelial cells.

### Goblet cell quantification

The number of goblet cells was assessed on PAS stained tissue sections. A total of 118 airways from 28 animals representing animals from the different exposure times was analyzed and cells were expressed as cell numbers per mm of P_BM_.

### Semiquantitative assessment of collagen deposition

To address whether chlorine exposure could affect the development of subepithelial fibrosis, lung sections were stained with Picrosirius red and collagen deposition scored in airways. Scoring by two blinded observers of collagen deposition in airways was performed independently using a scale from 1 to 3. The cumulative score for each mouse was averaged according to treatment group.

The quantity of airway smooth muscle (ASM) was quantified by the *camera lucida *technique. Images of the airways were traced using a microscope side arm attachment and areas of the α-actin positive smooth muscle bundles were digitized using commercial software. The area of ASM was standardized for airway size using the P_BM_, with the quantity of ASM expressed as ASM/P_BM_^2 ^(mm^2^). Morphometric assessments were made on all airways in the tissue section that met the above criterion for its aspect ratio.

### Methacholine responsiveness

In a separate group of sixty mice, airway responsiveness to methacholine was measured at similar time points after room air or Cl_2 _exposure (n = 10 at each time point). Animals were sedated with xylazine hydrochloride (10 mg/kg i.p.) and anaesthetized with sodium pentobarbital (40 mg/kg i.p). A flexible, saline-filled cannula (PE-10 tubing) was inserted into the jugular vein for administration of drugs and the trachea was cannulated with a snug-fitting metal cannula. Animals were connected to a computer-controlled small animal ventilator (flexiVent, Scireq, Montreal, PQ, Canada) and paralysed using pancuronium chloride (0.8 mg/kg i.v.). Mice were ventilated in a quasi-sinusoidal fashion with a tidal volume of 0.18 ml at a rate of 150 breaths/min. A positive end-expiratory pressure (PEEP) of 1.5 cmH_2_O was used. Measurements of pulmonary mechanics were made using a 2.5 Hz sinusoidal forcing function with an amplitude of 0.18 ml. The perturbation was applied after cessation of regular ventilation and expiration by the animal to functional residual capacity. Respiratory system resistance (Rrs) and dynamic elastance (Ers) was derived from the relationship between airway opening pressure, tidal flow and volume After initial baseline measurements of Rrs and Ers, doubling doses of methacholine chloride (Sigma;10 μg/kg to 320 μg/kg i.v.) were administered. Rrs and Ers were measured every 15 seconds after methacholine infusion until peak Rrs was reached. Thirty seconds after peak Rrs was reached, the next highest dose of methacholine was administered. The peak Rrs and Ers at each methacholine dose were used to construct a dose-response curve. After completion of all methacholine doses, animals were euthanized by i.v. pentobarbital overdose. Airway responses were evaluated as the difference between the peak in Ers after 160 μg/kg methacholine and baseline Ers (ΔErs). Changes in Ers rather than Rrs were chosen to represent airway responsiveness because methacholine-induced changes in elastance are affected to a greater degree in mice after Cl_2 _exposure[9].

### Statistical analysis

One-way analysis of variance was used to determine the effect of time on the dependent variables except ASM/mm^2^. The significance of the post-hoc comparisons was determined using Dunnett's test versus control at the p < 0.05 level. The effect of Cl_2 _on ASM/P_BM_^2 ^(in mm^2^) at different times after exposure was tested using the Kolmogorov-Smirnoff test.

## Results

### Histological and immunohistochemical evaluation of airways

**Figure 1:**
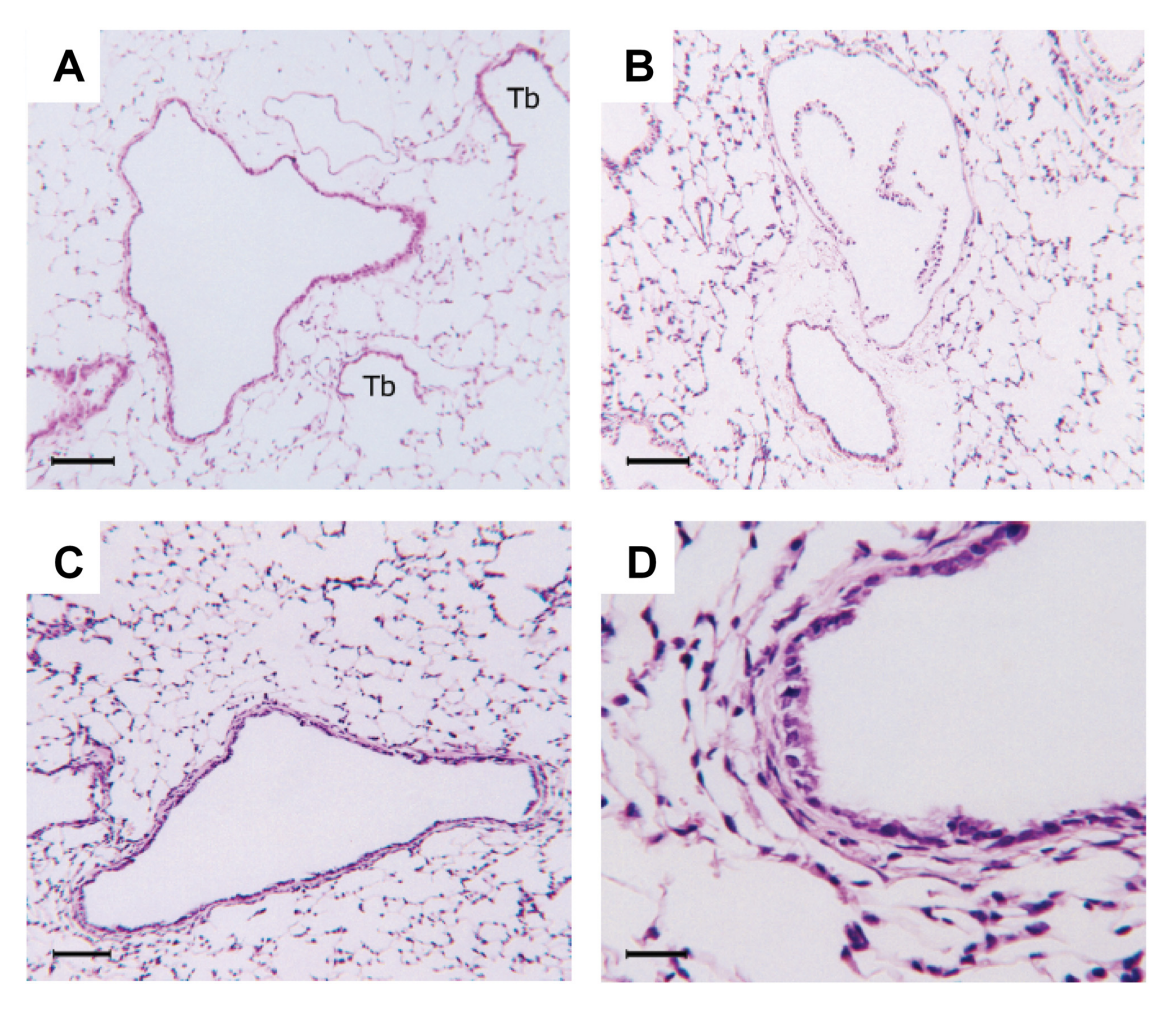
Effects of Cl_2 _exposure on lung histology. A: Normal mouse lung showing a large airway in cross section, an accompanying artery and two terminal bronchioles (Tb) that open into their respective alveolar ducts. B: Lung histology 12 h after a single 800 ppm Cl_2 _exposure. Partial or complete detachment of airway epithelium, as seen in this example, occurred in all airways. C: 10 d post-exposure, the epithelium is reconstituted and the airway wall is thickened. D: 10 d post-exposure, high magnification detail showing fully reconstituted airway epithelium. Stain: H&E. Scale bars: 100 μm in A-C; 25 μm in D.

**Figure 2:**
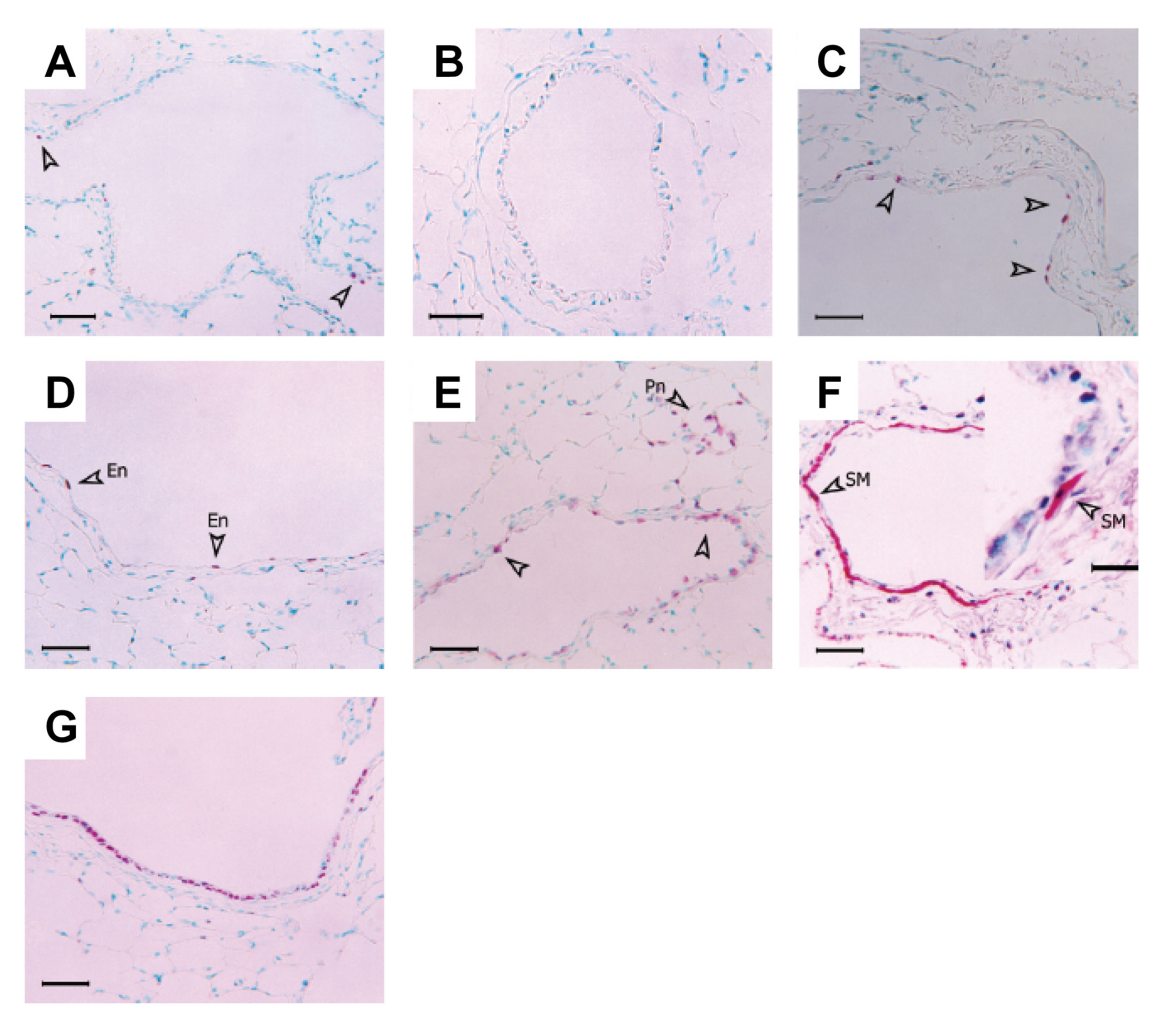
Effect of Cl_2 _exposure on cell proliferation as detected by PCNA immunostaining. A: Control mouse airway, showing baseline airway epithelial cell proliferation. PCNA positive cells are indicated by open arrowheads. B: 12 h post-exposure. There is an absence of PCNA positive events, suggesting inhibition of cell cycle. C and D: 24 h post-exposure. Proliferation of airway epithelial cells (C) is re-established. Endothelial cell proliferation (En) is also observed at this time point (D). E: 48 h post-exposure. An increase in PCNA positive epithelial cells is observed. F: Co-localisation of smooth muscle α-actin (red cytoplasmic signal) and PCNA (dark-violet nuclear signal), 48 h post-exposure. PCNA positive cells can be seen in the airway epithelium, smooth muscle layer, and adventitia. The inset shows an example of a PCNA positive airway myocyte at high magnification. G: 5 d post-exposure. The airway epithelium is evenly re-populated with cells undergoing intense proliferative activity. Scale bars: 50 μm (25 μ in F inset). Pn: Pneumocytes; SM: Smooth muscle.

**Figure 3:**
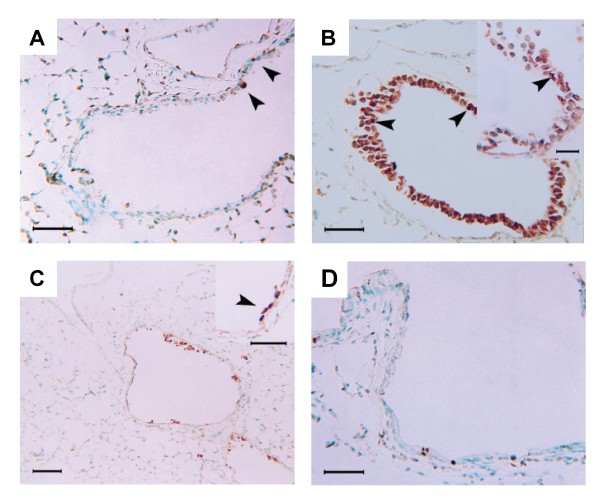
Effect of Cl_2 _exposure on airway cell apoptosis; TUNEL technique. A: Control mouse airway, showing baseline airway epithelial cell apoptosis (arrowheads). B: 12 h post-exposure. Cytoplasmic TUNEL signal in damaged epithelium. The high magnification inset details the cytoplasmic localisation of the TUNEL stain on cells with methyl green counterstained nuclei. These cells lack a TUNEL signal attributable to apoptosis-related DNA fragmentation. The arrowheads indicate examples of cells that appear truly apoptotic. C: 24 h post-exposure. Some clusters of basal cells undergoing apoptosis are visible. Inset shows high magnification detail. D: 5 d post-exposure. The frequency of TUNEL positive cells at 5 d is back to baseline level. Scale bars: 100 μm in I; 50 μm in A, B, C inset and D.

Normal airway structure and basal levels of proliferation and apoptosis in airway epithelium are shown in Figures 1A, 2A, 3A. Histological examination from samples obtained 12 hrs after exposure showed severe injury to the bronchial epithelium with extensive detachment of the epithelium from the basement membrane and complete denudation of the epithelium in some airways (Figure 1B). Cell cycle was inhibited at this time point after chlorine exposure, as indicated by the virtual absence of positive staining for PCNA (Figure 2B). The TUNEL technique produced cytoplasmic staining of the injured epithelium, but not a signal conforming to usual histopathological criteria for the identification of apoptosis, suggesting that a mechanism other than apoptosis accounts for the rapid and massive epithelial disaggregation following Cl_2 _gas exposure (Figure 3B). At 24 hrs after Cl_2 _exposure, most of the detached airway epithelial cells were cleared and airway epithelial cell proliferation was re-established (Figure 3C). In this phase, some clusters of basal cells undergoing apoptosis alternated with proliferating cells, overlying a preserved basement membrane (Figure 3D). Epithelial regeneration was evident at 48 hrs with flattened cells with elongated nuclei lining the basement membrane and an increased frequency of PCNA positive cells. Co-localisation of PCNA and smooth muscle α-actin provided evidence of airway smooth muscle proliferation (Figure 2F). Five days following chlorine exposure, the airway epithelium was evenly re-populated with cells showing an intense proliferative activity, and the frequency of apoptotic cells was similar to baseline levels. Ten days after chlorine exposure, the epithelium was reconstituted and the airway wall was thickened (1 D). Cl_2 _exposure did not induce goblet cell metaplasia as determined by PAS staining at any time point (data not shown). Only 4 of 118 airways analyzed from 28 mice, sampled at all time points showed any PAS positive cells and these were very infrequent.

**Figure 4:**
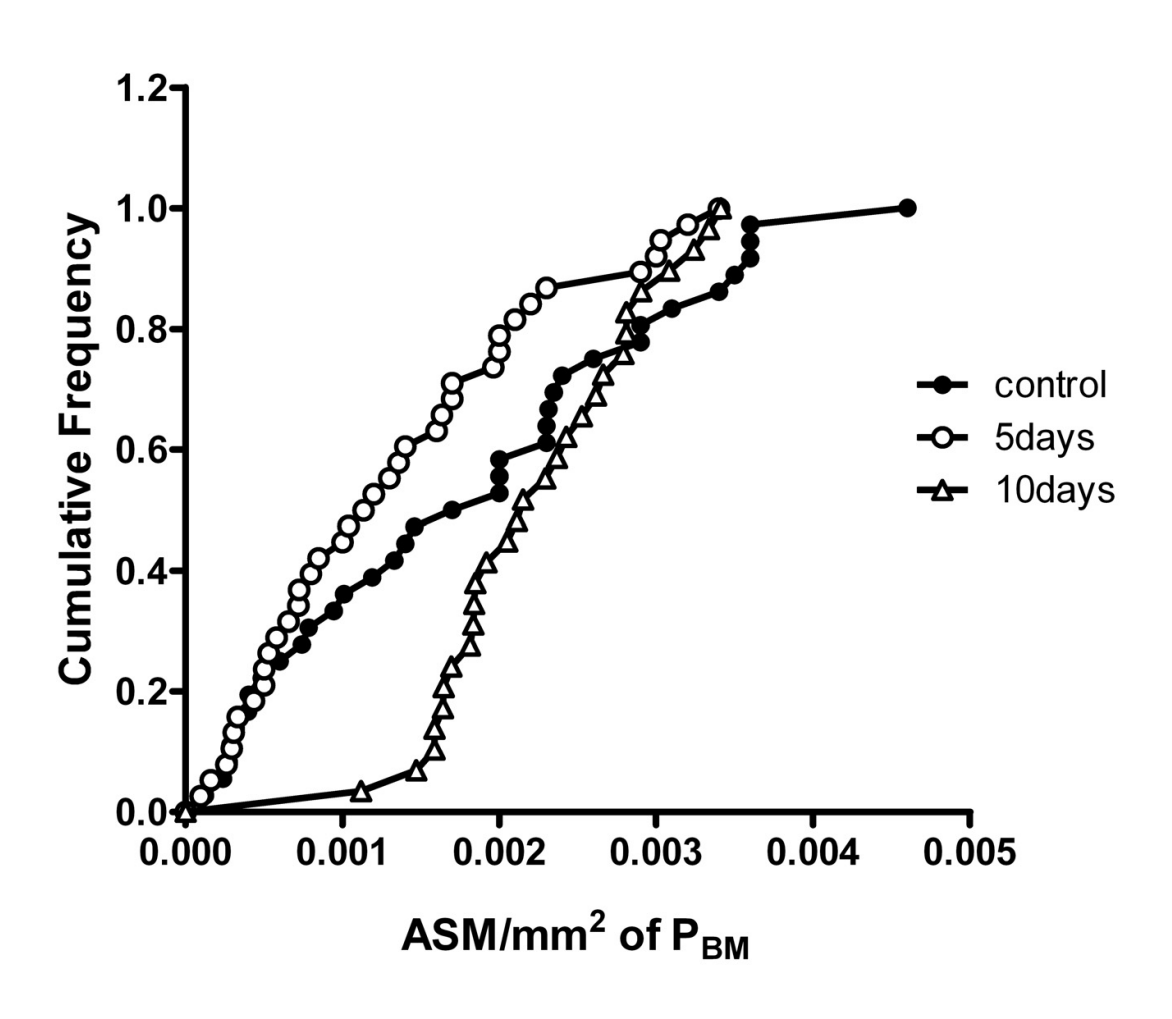
Cumulative distribution of airway smooth muscle mass per mm^2 ^of basement membrane (ASM/mm^2 ^of P_BM_). The values plotted are individual airway measurements. 2–8 airways were quantified per animal. The distribution of the 10 day group was significantly different from both the control and 5 day groups (p < 0.05). n = 38, 40, and 31 for control, 5 days, and 10 days.

Cl_2 _exposure did affect the quantity of ASM as determined by morphometry (Figure 4). 10 days after Cl_2 _exposure, a shift was observed in the distribution of airways with small amounts of ASM. For example, the proportion of airways with values of ASM area > 0.0015 (ASM/mm^2 ^of BM) was approximately 50% for control animals, but < 10% for the 10 day post-exposure group.

### Quantification of PCNA

**Figure 5:**
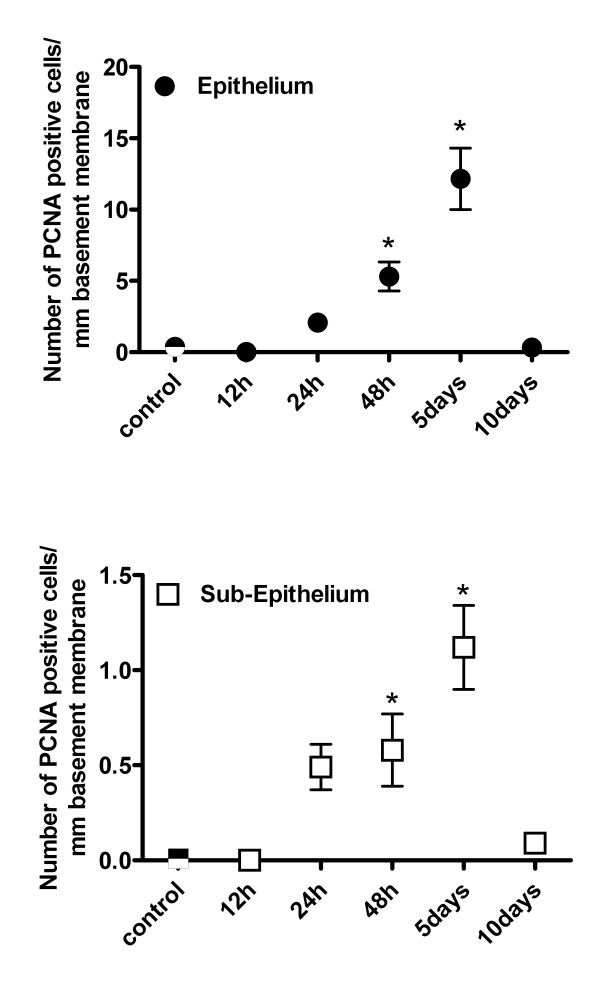
Time course of PCNA expression in the epithelium (A) and subepithelium (B) of airways in mice exposed to air (control) or Cl_2 _gas. Data is expressed as PCNA-positive cells/mm basement membrane. The number of airways evaluated at each time point ranged from 25 to 57. Values are means ± S.E. *significantly different from control (p < 0.05).

The number of PCNA+ cells in the airway epithelium and sub-epithelium is shown in Figure 5. A baseline frequency of epithelial and sub-epithelial proliferation was detectable in control animals. Twelve hours after Cl_2 _exposure, epithelial PCNA expression tended to be lower than control values although the difference did not reach statistical significance. Epithelial PCNA expression was significantly elevated by 48 hrs after chlorine exposure, increasing approximately 14-fold from control levels (p < 0.05) and over 30-fold by 5 d post-exposure (p < 0.05). Although the majority of the PCNA+ cells in the airways were epithelial cells, a significant amount of sub-epithelial PCNA expression was also observed after Cl_2 _exposure. Subepithelial PCNA expression was significantly elevated at 5 d post-exposure. By 10 d post-exposure, both epithelial and subepithelial PCNA immunoreactivity had returned to control levels. No significant correlation was found between airway size (as determined by basement membrane length) and PCNA index at any of the time points.

### Determination of airway fibrosis

**Figure 6:**
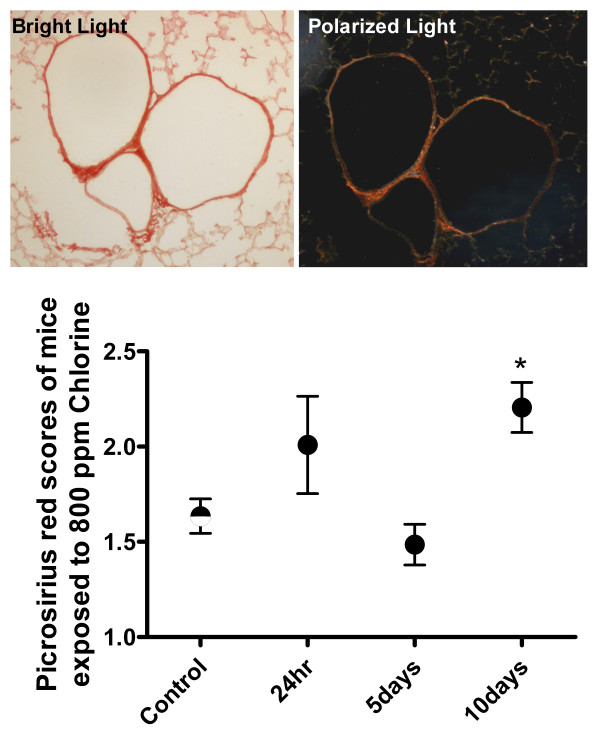
Illustrative photomicrograph showing collagen in the airway walls by Picrosirius red staining (two left panels). Quantitative analysis of degree of staining by semi-quantitative scoring at different time points after Cl_2 _gas exposure.

Assessment of collagen deposition using Picrosirius red staining demonstrated a significant increase in collagen in the airways 10 days following chlorine exposure (Figure 6). There was no significant difference in the amount of collagen at 24 hours or 5 days. Twenty nine animals were analyzed and assessed by two observers independently.

### Bronchoalveolar lavage

**Figure 7:**
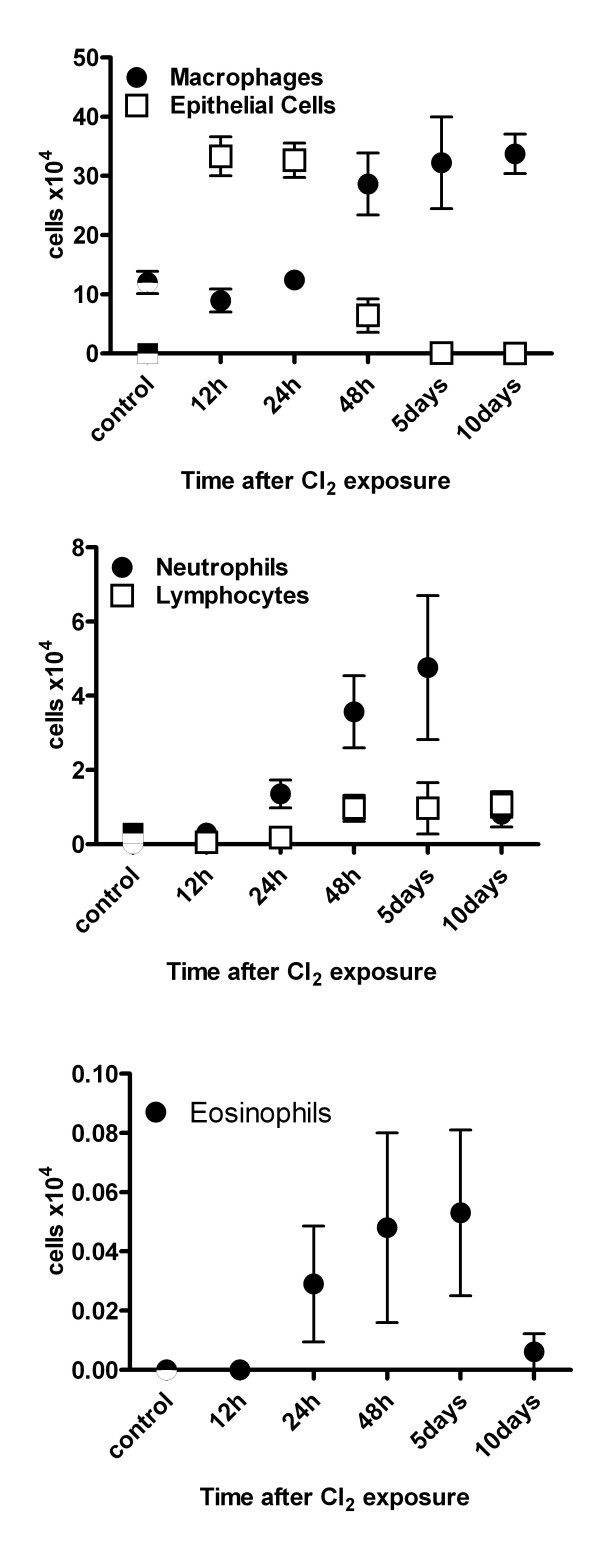
Time course of BALF differential cell counts after a single Cl_2 _gas exposure. At each time point, n = 8. Values are means ± S.E. * significantly different from control (p < 0.05).

The recovery of BALF averaged 90% and did not differ significantly among groups. Total cell counts were significantly elevated at 5 d and remained elevated at 10 d post-exposure relative to controls (Table 1). Differential cell counts showed no significant change in eosinophils or lymphocytes after Cl_2 _exposure (Figure 7), but neutrophils were significantly elevated relative to controls at 5 d post-exposure (0.02 ± 0.01 (SE) × 10^4 ^cells in controls, 4.76 ± 1.94 at 5 d post-exposure; p < 0.05) and macrophages were significantly elevated at both 5 d and 10 d post-exposure (12.0 ± 1.9 × 10^4 ^in controls, 32.2 ± 7.7 at 5 d, 33.7 ± 3.3 at 10 d, p < 0.05 versus controls). Dead cells in the BALF, identified by trypan blue, were markedly elevated from 12 hrs to 48 hrs post-exposure (Table 1); these cells were almost exclusively comprised of epithelial cells, identified by their cuboidal shape and cilia. Similarly, the number of epithelial cells counted during differential cell counting of cytospin slides was markedly elevated at 12 and 24 hr (p < 0.05) but had returned to control levels by 48 hr (Figure 7). The amount of total protein in BALF supernatant, a marker of airway microvascular permeability and epithelial damage, was significantly elevated 12 hrs after chlorine exposure, and remained elevated up to 5 d post-exposure (Table 1).

**Table 1:** Time course of protein, live and dead cell counts in BALF after Cl_2 _exposure.

	Control	12 hr	24 hr	48 hr	5 d	10 d
Live cells (×10^4^/ml BALF)	12.3 ± 1.9	9.4 ± 1.9	14.0 ± 1.1	33.1 ± 6.3	38.0 ± 9.8*	36.9 ± 3.4*
Dead cells (×10^4^/ml BALF)	1.3 ± 0.5	92.6 ± 11.2*	106.2 ± 9.7*	54.1 ± 17.0*	5.8 ± 0.7	3.5 ± 0.6
Protein (g/ml)	69.4 ± 8.1	612.8 ± 178.6*	391.7 ± 102.2*	251.5 ± 29.5*	221.0 ± 42.7*	116.7 ± 6.3

Values are means ± S.E. * p < 0.05 versus control

### Airway mechanics and responsiveness to methacholine

**Figure 8:**
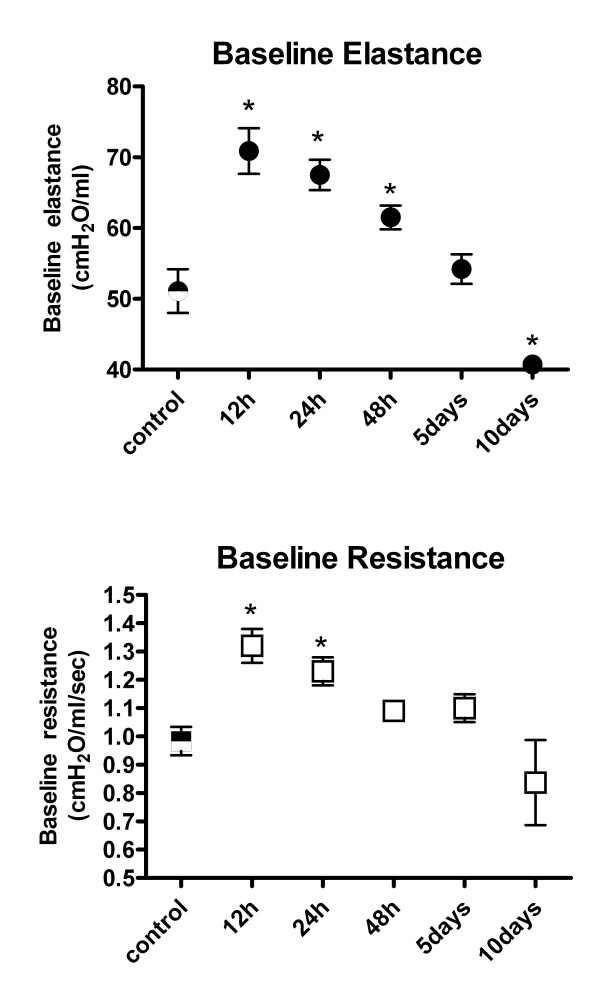
Time course of baseline respiratory elastance (Ers) and resistance (Rrs) in mice exposed to Cl_2 _gas. Ers and Rrs were measured using a 2.5 Hz sine-wave perturbation with an amplitude of 0.18 ml. At each time point, n = 10. Values are means ± S.E. * significantly different from control (p < 0.05).

**Figure 9:**
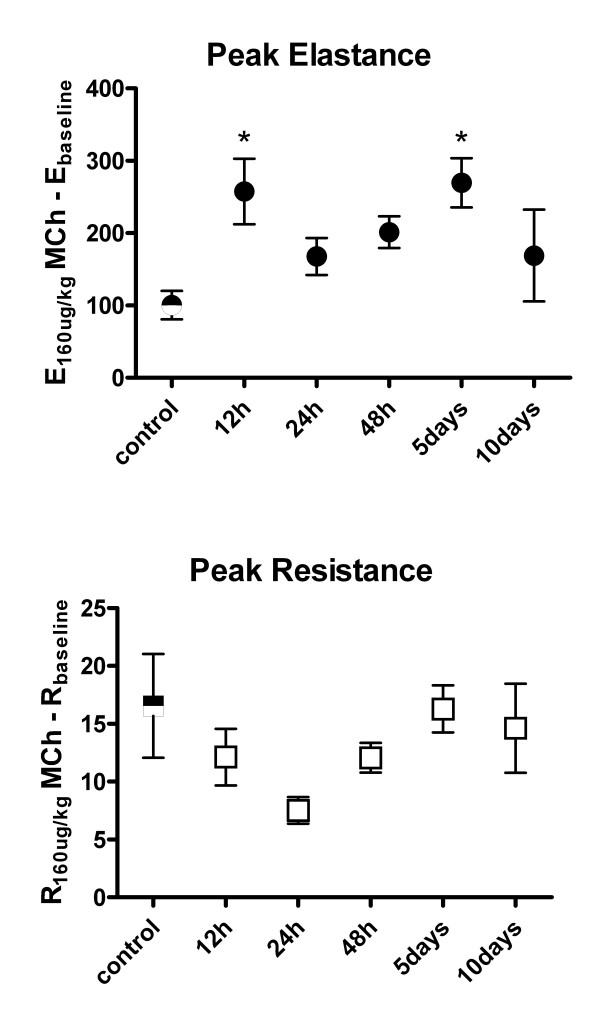
Time course of airway responsiveness of elastance (Ers) and resistance (Rrs) to methacholine in mice exposed to Cl_2 _gas. Responsiveness is expressed as the peak Ers or Rrs after administration of 160 μg/kg methacholine minus baseline Ers or Rrs. Values are means ± S.E. * significantly different from control (p < 0.05).

Cl_2 _exposure altered respiratory mechanics as reflected by changes in baseline Ers and Rrs. The initial response to Cl_2 _exposure was an elevation of Ers and Rrs, which persisted up to 48 hrs post-exposure (Ers = 51.1 ± 3.09 cmH_2_O/ml in control mice vs. 70.9 ± 3.23, 67.5 ± 2.16, and 61.5 ± 1.67 cmH_2_O/ml at 12, 24, and 48 hrs post-exposure respectively, p < 0.05; Rrs = 0.98 ± 0.05 cmH_2_O/ml/sec in control mice vs. 1.32 ± 0.06 and 1.23 ± 0.05 cmH_2_O/ml/sec at 12 and 24 hrs post-exposure respectively, p < 0.05) (Figure 8). Airway mechanics returned to baseline levels by 5 d, but at 10 d post-exposure, Ers levels fell significantly below control levels (Ers = 51.1 ± 3.09 cmH_2_O/ml in control mice vs. 40.7 ± 0.97 cmH_2_O/ml at 10 d post-exposure, p < 0.05). Airway responsiveness to methacholine, as determined by ΔErs, increased after Cl_2 _exposure compared to control, and was significantly higher at 12 hrs and 5 d post exposure (ΔErs = 100 ± 19.7 in control mice vs. 257 ± 45.3 and 269 ± 34.0 at 12 hrs and 5 d post-exposure respectively, p < 0.05) (Figure 9). ΔRrs was not significantly altered at any time point after Cl_2 _exposure, although a trend for ΔRrs to be lower 24 hrs after Cl_2 _exposure was observed (p = 0.055).

## Discussion

This study describes the time course of airway epithelial damage and repair in A/J mice following a single exposure to a high concentration of Cl_2 _gas. Cl_2 _exposure resulted in marked damage to the airways, as indicated by epithelial cell sloughing, increased protein in BALF, an inflammatory response with neutrophil and macrophage recruitment into the airways, and altered lung mechanics. Subsequent airway repair was characterized by increased epithelial and subepithelial cell proliferation, complete restoration of the epithelial layer, increases in the quantity of ASM and modest airway hyperresponsiveness. There was also evidence of airway fibrosis at 10 days after the Cl_2 _exposure.

A pronounced feature of the acute injury phase after Cl_2 _exposure was extensive and synchronous loss of airway epithelial cells. Programmed cell death was not likely the mechanism of the generalized loss of epithelial cells, since the TUNEL technique did not produce a nuclear signal consistent with apoptosis. The explanation for the diffuse cytoplasmic staining observed in the detached epithelium is not clear but may have been caused by the highly reactive chlorine molecules. As opposed to apoptosis, disruption of the intercellular junctions and the attachments of the epithelial cells to the basement membrane by the Cl_2 _gas may have been the mechanism responsible for detachment of the epithelium. Other oxidants such as hypochlorous acid (HOCl) and ozone can disrupt cell adhesion via damage to extracellular matrix proteins and β-1 integrins[12,13], thus Cl_2 _gas may act via similar mechanisms.

Acute loss of epithelial barrier function resulted from the extensive sloughing of the airway epithelium, as reflected by the increased protein concentration in BALF. Changes in baseline respiratory mechanics (resistance and dynamic elastance) paralleled the time course of BALF protein concentration with the most pronounced alterations occurring 12 hrs after exposure followed by resolution of these changes over the 10 d study period. Pulmonary edema and alveolar flooding may have contributed to the acute decreases in lung elastance in this model, as has been demonstrated in other species after Cl_2 _gas exposure[14,15]. However, heterogeneous airway narrowing may have also contributed.

Exposure to chlorine gas exposure had a direct toxic effect on airway epithelium as severe airway damage was observed at early time points in the absence of an inflammatory response. When inhaled, chlorine gas combines with water to form hydrochloric and hypochlorous acids (Cl_2 _+ H_2_O → HCl + HOCl). HOCl is unstable and breaks down into HCl and free oxygen. Oxidant injury due to this nascent oxygen is thought to be the primary mechanism of cytotoxicity, with the acid production being secondary. In a similar study from our laboratory, positive staining for 3-nitrotyrosine residues, a marker of oxidative stress, was observed in mouse airways 24 hrs after exposure to 800 ppm Cl_2 _gas, supporting oxidative injury as a mechanism in this model[9].

A modest neutrophil and macrophage inflammation did subsequently develop after Cl_2 _exposure and the inflammatory cells themselves could also have contributed to airway damage. Activated neutrophils can produce reactive oxygen species and myeloperoxidase, a neutrophil-specific enzyme that catalyses the formation of hypochlorous acid/hypochloride (HOCl/OCl^-^) from hydrogen peroxide. Neutrophils can also release proteolytic enzymes such as collagenase and elastase which could also contribute to the airway damage.

Following the acute airway injury induced by Cl_2 _gas exposure, tissue repair and restoration of the barrier function of the epithelium occurred. One mechanism by which an epithelial layer can be repaired is by migration of healthy epithelial cells from an area adjacent to the damaged epithelium. Studies of mechanical de-epithelialisation *in vivo *demonstrate that this is a quickly occurring process, with initial migration of adjacent epithelium to the wound site occurring within 8–15 hrs[16]. The relevance of migration as a mechanism, however, is questionable in cases of near to complete denudation of the epithelium, as was observed in many airways in this study. In this instance, growth and differentiation of local progenitor cells is another mechanism by which the epithelial layer can be repopulated. In the trachea and bronchi, basal cells constitute a separate layer of cells attached to the airway basement membrane. In response to epithelial injury, these cells can turn into a highly proliferative cell phenotype and can become flattened and cover the basement membrane[17]. In smaller bronchioles, Clara cells likely play the role of progenitor cell after injury[18] Intriguing new evidence suggests a possible role for circulating bone marrow stem cells in bronchiolar repopulation after injury[19]. Ortiz et al. [19] have demonstrated that murine mesenchymal stem cells are able to home to the lung after injury and adopt an epithelium-like phenotype. It is uncertain at this time as to which specific cell population may have acted as progenitor cells for the airway epithelium in this study.

The time course of epithelial repair after Cl_2 _gas exposure was assessed by quantifying the amount of cellular proliferation occurring in the airway. Increased levels of PCNA immunoreactivity were detectable by 48 hrs and maximal proliferative activity in the airways occurred 5 d post-exposure. Compared to other studies reporting dynamics of epithelial repair after acute airway injury, the recovery of murine airways from Cl_2 _damage was relatively prolonged. In rats, peak cell proliferation occurred 26 to 36 hrs after mechanical injury of tracheal epithelium[20,21] and at 24 to 48 hrs after acute ozone exposure[22]. In mice, epithelial cell proliferation after desquamation of airway epithelium by naphthalene treatment was maximal 2 to 7 days post-treatment depending on mouse strain[23]. The time course of epithelial repair after damage is likely related to the severity of injury, and therefore is difficult to compare among these different models.

Increased cellular proliferation after Cl_2 _exposure was not limited to the airway epithelium as significant PCNA immunoreactivity was also observed in the sub-epithelial layer of airways. Using immunohistochemical co-localization, we provide evidence of airway smooth muscle cell proliferation. This finding, together with the quantification of ASM mass, suggests that chlorine exposure in this model results in ASM hyperplasia. This is in agreement with the study of Demnati et al [8] who reported an increase, albeit transient, in ASM quantity in rats after acute exposure to Cl_2 _gas.

The signals involved in repair and in the repopulation of the epithelium after Cl_2_-induced injury are unclear. Epidermal growth factor (EGF)-dependent mechanisms may be important as mediators such as epidermal growth factor (EGF) and TGF-α can bind to EGF receptors located on both basal cells and epithelial cells and stimulate cell migration, proliferation and differentiation[24]. The absence of goblet cells is however somewhat surprising if indeed EGF receptor ligands are important in repair as stimulation of the EGF receptor has been repeatedly demonstrated to cause goblet cell differentiation in the airways[25]. EGF-independent factors may also be important. Neutrophils, for example, may contribute to signalling of repair processes as neutrophil defensins, antimicrobial peptides present in the neutrophil, may also stimulate proliferation[26]. Interestingly, the maximal proliferative activity of the airway epithelium at 5 d corresponded to the time of maximal neutrophil influx in the BALF.

Restoration of the airway epithelial layer, as assessed histologically, was complete by 10 days after Cl_2 _exposure. However, not all variables had returned to control levels after 10 days; inflammatory cells in the BALF were still elevated and baseline elastance was lower than control levels. Therefore complete resolution of the Cl_2_-induced damage may not have occurred in the timeframe of this study. Also the timeframe of this study may not have been long enough to fully evaluate remodelling processes. As we only detected changes in ASM quantity at our latest time point, 10 days after exposure, the possibility remains that further remodelling may take place at even later time points. There was also an increase in collagen deposition in the airway wall at this same time point. The epithelium is a source of fibrogenic cytokines[27] and it is potentially the cause of the collagen deposition. Although the changes were not significant there appeared to be a trend for a reduction in airway smooth muscle mass at 5 days after Cl_2 _exposure, suggesting that damage may have penetrated beyond the epithelium to the ASM layer.

Persistent airway hyperresponsiveness occurs in a small percentage of people after acute Cl_2 _gas exposure[28]. In this study, mice receiving a single exposure to a high concentration of Cl_2 _gas did display modest increase in dynamic elastance in response to methacholine but it was transient in nature. That responsiveness of pulmonary dynamic elastance to methacholine was affected to a greater degree by Cl_2 _gas exposure than was responsiveness of pulmonary resistance is consistent with results from a previous study[9]. This suggests that changes in responsiveness to methacholine after Cl_2 _gas exposure in mice may be dominated by abnormalities in the peripheral lung, as opposed to central airways. Perhaps also the trend for a reduction in responsiveness to methacholine may reflect injury to the airway smooth muscle from the high levels of Cl_2 _used for exposure.

In conclusion, this study describes the time-course of injury and repair after an acute exposure of mice to a high concentration of Cl_2 _gas. Severe epithelial injury was induced quickly after exposure with loss of the epithelial barrier function and acute alterations in respiratory mechanics. Epithelial repair processes were apparent by 24 hrs and restoration of the epithelium was complete by 10 d. Recovery from the Cl_2_-induced damage was associated with modest airway hyperresponsiveness and alterations in airway smooth muscle mass. Whether comparable airway remodelling is associated with lesser degrees of repeated exposures remains to be explored.

## Competing interests

The authors declare that they have no competing interests.

## Authors' contributions

ST was involved in the design and performance of the experiments and wrote the manuscript. DRB was responsible for the planning and oversight of all immunohistochemistry and contributed to the manuscript. HC assisted in the performance of measurements of airway responsiveness and tissue harvesting. TM performed histochemical staining for goblet cells and collagen and performed quantification of same. HKQ assisted in the analysis of histochemical images for goblet cells and collagen and assisted in editing the manuscript. JGM was responsible for the questions being tested and for the design of the experiments. He reviewed all phases of analysis and finalized the writing of the manuscript. All of the authors have read and approved the manuscript.
